# Electrospun Core–Shell Bioadhesive Nanofiber Sheets Enable Durable Pulmonary Air Leak Sealing Under Wet and Dynamic Conditions

**DOI:** 10.1002/adhm.71789

**Published:** 2026-10-08

**Authors:** Tomohito Okubo, Kouji Hara, Naoyuki Hatayama, Kota Takahashi, Eriko Yamazoe, Takaaki Ito, Takao Takeuchi, Yoko Koide, Mayumi Yamada, Munekazu Naito, Kohei Tahara

**Affiliations:** ^1^ Department of Anatomy Aichi Medical University Nagakute Aichi Japan; ^2^ Food & Healthcare Incubation Department Mitsubishi Chemical Corporation Chiyoda Tokyo Japan; ^3^ Laboratory of Nanofiber Technology Gifu Pharmaceutical University Gifu Japan; ^4^ Laboratory of Pharmaceutical Engineering Gifu Pharmaceutical University Gifu Japan

**Keywords:** bioadhesive material, core–shell structure, electrospinning, nanofiber sheet, pulmonary air leak, thoracic surgery

## Abstract

Pulmonary air leak is the most common complication following lung surgery. Most materials used for pulmonary fistula closure rely on fibrin network‐mediated adhesion. However, on the moist and dynamically deforming lung surface, these materials often fail to provide sufficient adhesion, and postoperative air leaks remain a major clinical concern. Since these materials are biologically derived, concerns regarding biosafety risks and high production costs persist. To address these limitations, this study develops a novel closure material that integrates biocompatibility, pressure resistance, tissue adhesion, and seal retention under wet conditions. The closure material is fabricated as a nanofiber sheet via electrospinning. Comparative evaluation of different structural designs demonstrates that incorporating a core–shell nanofiber structure achieves a favorable balance of pressure resistance, tissue adhesion, and seal retention under wet conditions. Acute leak closure was evaluated in five animals, and long‐term safety was assessed in three animals followed for 1 month. The mean air leak volume decreased from 46.2 to 1.8 mL/min, with post‐treatment air leak volumes below the clinically accepted threshold (≤ 10 mL/min) in all five animals. No treatment‐related complications were observed during the follow‐up period. Thus, electrospun nanofiber sheets represent a promising and biosafe approach for pulmonary air leak closure.

## Graphical abstract

A core–shell electrospun nanofiber sheet provides strong adhesion and durable sealing on the wet and dynamic lung surface. The engineered fibrous architecture maintains airtight integrity and offers a potential alternative to fibrin sealants for pulmonary air leak management in thoracic surgery.

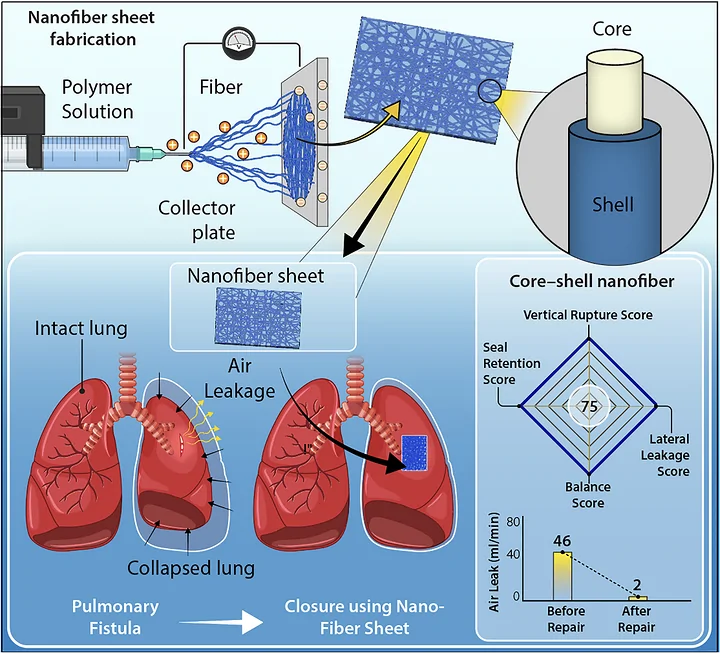

## 1 Introduction

Pulmonary air leaks are among the most common complications in pulmonary surgery and are a major clinical concern. Globally, the number of individuals living with chronic obstructive pulmonary disease has increased substantially over the past three decades [1]. Chronic obstructive pulmonary disease is characterized by fragile lung tissue resulting from chronic airway inflammation [2]. Tissue fragility is associated with a markedly high incidence of complications, such as alveolar–pleural fistula and bronchopleural fistula, both of which cause prolonged air leak [3]. Prolonged air leak considerably impairs patients’ quality of life and negatively impacts overall prognosis [4]. In addition, tissue fragility makes surgical repair using conventional suturing techniques particularly challenging. Thus, there exists a growing demand for lung repair strategies that utilize tissue reinforcement materials capable of achieving high airtightness without reliance on conventional suturing techniques.

Biologically derived fibrin‐based products are the most commonly used reinforcement materials for pulmonary air leak repair [5]. However, concerns regarding insufficient sealing strength, biosafety, and manufacturing costs have motivated the development of alternative sealing materials, including lung‐mimetic hydrofoams and photocrosslinkable hydrogels [6, 7, 8, 9, 10]. Although these approaches have shown promising sealing performance, a material that simultaneously satisfies the diverse requirements for pulmonary fistula closure has yet to be established.

The lung surface represents a mechanically dynamic and constantly moist environment that imposes unique demands on sealing materials. Accordingly, effective pulmonary fistula closure requires materials that can rapidly adhere to irregular tissue surfaces, withstand airway pressure, and maintain stable sealing performance under wet and repetitive respiratory loading conditions. However, these properties are often difficult to achieve simultaneously within a single material system because enhancing one function may compromise another. We therefore hypothesized that a two‐layer treatment strategy, consisting of an adhesive inner layer and a mechanically durable outer layer, could provide a more effective solution for pulmonary fistula closure. To implement this strategy, a material platform capable of providing both biocompatibility and structural design flexibility is required.

Electrospun nanofiber sheets possess several biological and structural properties that make them attractive candidates for organ reinforcement materials. Their excellent cell adhesiveness and nutrient diffusivity support tissue regeneration, while their ability to prevent bacterial invasion, retain moisture, and serve as drug delivery platforms has led to their widespread use in regenerative medicine and wound management [11, 12, 13]. Moreover, the highly tunable nature of electrospinning enables precise control over fiber composition and architecture. By leveraging this versatility, we aimed to develop a reinforcement material capable of balancing tissue adhesion and seal durability under wet pulmonary conditions. Different electrospun architectures may provide distinct balances between adhesive performance, pressure resistance, and seal retention under wet conditions. Therefore, in this study, we systematically evaluated several nanofiber architectures to identify a design suitable for pulmonary fistula closure and to assess the effectiveness of a two‐layer treatment strategy.

## 2 Results

### 2.1 Study Design

This study aimed to evaluate the utility and clinical applicability of electrospun nanofiber sheets in pulmonary fistula closure. The therapeutic concept of these sheets is to achieve fistula closure and promote healing by applying the sheets directly to the injured lung surface (Figure 1A). Electrospinning was used as the core fabrication technique for sheet production (Figure 1B). Tables 1 and 2 summarize the compositions of the electrospinning solutions and the fabrication parameters, including applied voltage, flow rate, tip‐to‐collector distance, collector voltage, spinning time, and environmental conditions. We adopted a two‐layer treatment strategy and fabricated one type of first‐layer sheet and four types of second‐layer sheets. The first‐layer sheet was referred to as the base sheet, and the second‐layer sheets were designated according to their structural characteristics as hybrid‐polyvinyl alcohol (PVA) sheet, core–shell sheet, layer‐by‐layer sheet, and emulsion sheet.

**FIGURE 1:**
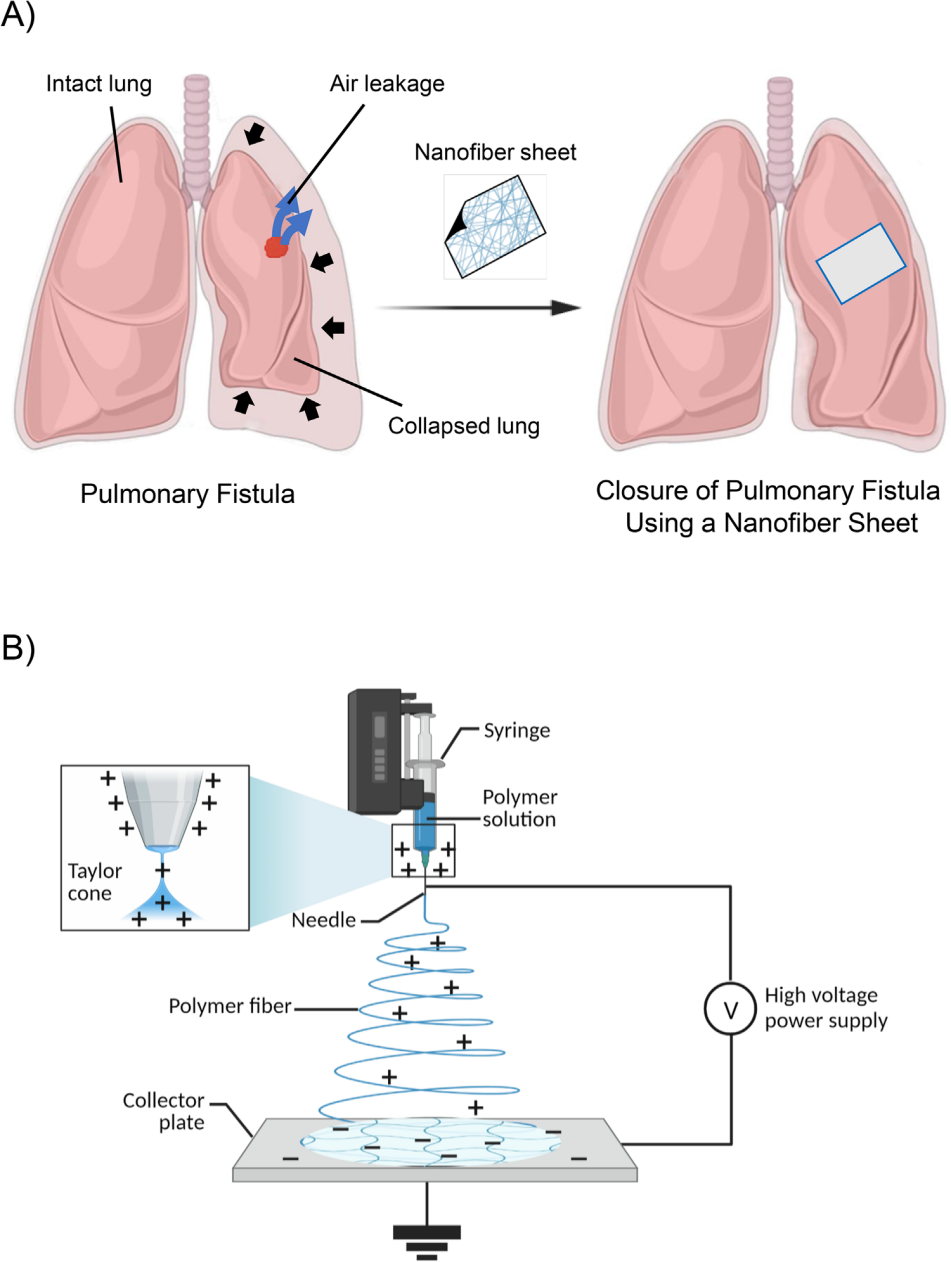
Schematic representation of the therapeutic concept and fabrication technique of nanofiber sheets. (A) Application of the sheet to the fistula site halts air leakage and promotes the spontaneous healing of the fistula. (B) Electrospinning method for the fabrication of nanofiber sheets. This figure was created using BioRender.com.

**TABLE 1:** Compositions of electrospinning solutions used for the fabrication of each nanofiber sheet type.

Sheet type	Solution role	Bioadhesive/water‐soluble polymer	Bioadhesive/water‐soluble polymer	Fully‐hydrolyzed PVA (water resistance)	Biodegradable/hydrophobic polymer	Solvent
		PVA EG‐40P (%)	CMC‐Na (%)	PVA NH‐26 (%)	PCL (%)	
Base	—	9.58	1.06	—	—	Water
Hybrid‐PVA	—	1.33	—	6.67	—	Water
Core–shell	Core solution	—	—	—	10.71	Chloroform/DMF (8:2)
	Shell solution	9.58	1.06	—	—	Water
Emulsion	—	4.72	0.53	—	4.72	Water/Ethyl acetate
Layer‐by‐layer	First‐layer solution	—	—	—	10.71	Chloroform/DMF (8:2)
	Second‐layer solution	9.58	1.06	—	—	Water

*Note*: For multi‐solution systems, such as the core–shell and layer‐by‐layer sheets, each individual solution (e.g., core/shell or first/second layer) is listed separately. Bioadhesive and water‐soluble polymers include PVA EG‐40P and sodium carboxymethyl cellulose (CMC‐Na). Fully hydrolyzed PVA (PVA NH‐26) was used to modulate water resistance. Poly(ε‐caprolactone) (PCL) was used as a biodegradable and hydrophobic polymer. Solvent systems were selected based on polymer solubility and processing compatibility.

**TABLE 2:** Electrospinning conditions used for the fabrication of each nanofiber sheet type.

Sheet type	Solution role	Needle voltage (kV)	Collector voltage (kV)	Tip‐to‐collector distance (cm)	Flow rate (mL/h)	Spinning time (min)
Base	—	21	−2	13	0.45	120
Hybrid‐PVA	—	17	−2	14	1	73
Core–shell	Shell solution	22	−8	16	0.55	110
	Core solution				0.11	
Emulsion	—	19.8	−2	14	0.8	90
Layer‐by‐layer	First‐layer solution	15.5	−2	13	0.5	24
	Second‐layer solution	16.5	−2	15	0.3	200

*Note*: For the base, hybrid‐PVA, and emulsion sheets, a single electrospinning solution was delivered through one syringe pump and electrospun in a single step. For the core–shell sheet, two different solutions were delivered simultaneously using separate syringe pumps connected to a coaxial nozzle, allowing the formation of a core–shell structured fiber in one continuous process. For the layer‐by‐layer sheet, the first layer was electrospun using one polymer solution. The solution was replaced with a second formulation to spin the second layer sequentially using the same electrospinning setup. Needle voltage, collector voltage, tip‐to‐collector distance, solution flow rate, and spinning time are summarized for each solution.

The study workflow was as follows. First, we characterized the physicochemical properties of each sheet, namely morphological features, mechanical properties, and water‐immersion stability. Next, we tested sealing performance using an animal pulmonary fistula model and compared the fistula closure efficacy of each sheet when applied to lung tissue using the two‐layer strategy. Sheet performance was evaluated using our original scoring system (Table S1). Based on the scoring results, the best‐performing sheet was selected for subsequent in vivo therapeutic evaluation using the pulmonary fistula animal model. Acute leak closure performance was first evaluated in a pilot study involving two dogs under closed‐chest conditions, after which the animals were euthanized at the end of the experiment. Following this preliminary evaluation, three dogs were enrolled in the main study and followed for 1 month postoperatively to assess the long‐term stability of fistula closure and in vivo safety. The pilot and main studies used the same pulmonary fistula model, core–shell sheet treatment, ventilatory conditions, air‐leak measurement method, and definition of successful closure. Therefore, acute leak closure data from all five animals were subsequently pooled for an exploratory analysis. This pooled analysis was not prespecified in the original study protocol and should therefore be considered exploratory, providing preliminary rather than confirmatory evidence. Long‐term survival and follow‐up assessments were limited to the three animals in the main study. The long‐term stability of fistula closure was assessed by chest radiography on postoperative days (PODs) 1, 3, 7, and 14. Chest radiographs were evaluated for evidence of pneumothorax, subcutaneous emphysema, and lung re‐expansion. Safety was assessed by blood tests on PODs 1, 3, 7, 14, and 28 and by histological evaluation of the treated site on POD 30. Blood tests included measurements of C‐reactive protein (CRP) and white blood cell (WBC) count as indicators of inflammatory responses, procalcitonin as a marker of infection, blood urea nitrogen (BUN) and creatinine to assess potential renal toxicity, and aspartate aminotransferase (AST), alanine aminotransferase (ALT), and gamma‐glutamyl transpeptidase (γ‐GTP) to assess potential hepatic toxicity. Histological evaluation was performed qualitatively and focused on inflammatory cell infiltration, foreign‐body giant‐cell formation, fibrosis, tissue integration, and tissue necrosis (Figure 2).

**FIGURE 2:**
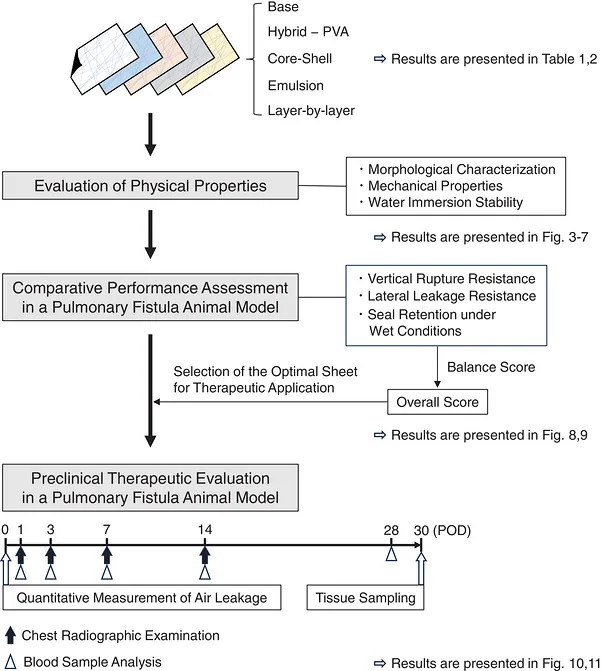
Experimental workflow for evaluating bioadhesive nanofiber sheets. One type of first‐layer sheet and four types of second‐layer sheets (base, hybrid‐polyvinyl alcohol [PVA], core–shell, emulsion, and layer‐by‐layer) were fabricated, and their morphological features, mechanical properties, and water immersion stability were evaluated. Performance was scored based on three parameters: vertical perforation resistance, side leakage resistance, and seal retention under wet conditions. Balance score and total performance score were derived using the three parameters. The optimal sheet was selected for a therapeutic study using a pulmonary fistula model in beagle dogs. Treatment efficacy and safety were assessed by measuring air leakage before and after treatment, along with postoperative radiographic imaging, blood tests, and histological evaluation.

### 2.2 Morphology of Electrospun Nanofiber Sheets

Across all sheet types, measured thicknesses were 60–100 µm. Representative scanning electron microscopy (SEM) images of the electrospun nanofiber sheets are shown in Figure 3. SEM images acquired at ×2000 magnification and used for the quantitative morphological analysis are provided in Figure S1.

**FIGURE 3:**
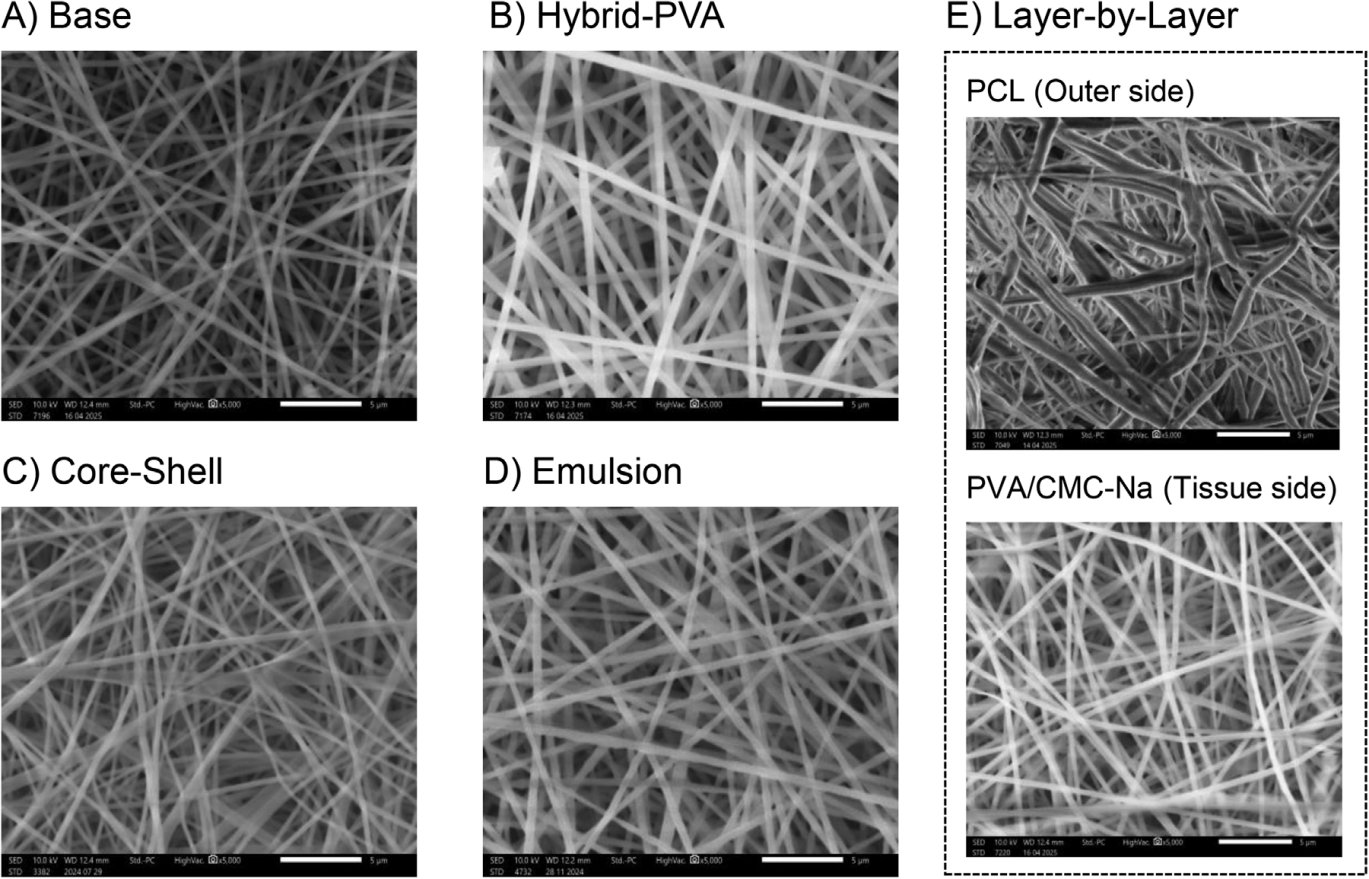
Scanning electron micrographs of electrospun nanofiber sheets: (A) base, (B) hybrid‐PVA, (C) core–shell, (D) emulsion, and (E) layer‐by‐layer (upper: PCL [outer side], lower: PVA/CMC‐Na [tissue side]). All sheet types consisted of continuous fibers without bead formation. Scale bars: 5 µm.

All sheet types exhibited continuous fibers without beads, confirming the successful electrospinning of nanoscale fibrous structures. Image analysis of SEM images using MIPAR software revealed that the mean fiber diameters ranged from 429 ± 190 to 666 ± 369 nm (Table S2). The base, core–shell, and emulsion sheets exhibited comparable mean fiber diameters (445 ± 179, 429 ± 190, and 446 ± 226 nm, respectively), whereas the hybrid‐PVA sheet and the PCL outer layer of the layer‐by‐layer sheet showed relatively larger fiber diameters (567 ± 209 and 666 ± 369 nm, respectively). The PVA/CMC‐Na tissue‐contacting layer of the layer‐by‐layer sheet exhibited a mean fiber diameter of 459 ± 184 nm.

Quantification of the segmented SEM images further demonstrated that the fiber and pore area fractions were comparable among the different sheet types. Fiber area fractions ranged from 49.6% to 54.5%, corresponding to pore area fractions of 45.5%–50.4% (Figure S2 and Table S2), indicating no marked differences in the overall porosity among the electrospun sheets.

Morphologically, the base and hybrid‐PVA sheets consisted of straight fibers with smooth surfaces. The core–shell sheet contained both relatively thin and thick fibers within the same fibrous mat, whereas the emulsion sheet exhibited slightly roughened fiber surfaces, likely reflecting the heterogeneous polymer distribution generated during emulsion electrospinning. The layer‐by‐layer sheet comprised two distinct fiber layers: a PVA/CMC‐Na tissue‐contacting layer with a morphology similar to that of the base sheet and a PCL outer layer consisting of relatively thicker fibers.

### 2.3 Internal Structure of Core–Shell and Emulsion Nanofibers

Transmission electron microscopy (TEM) analysis was performed only for the core–shell and emulsion nanofiber sheets because the other types (base, hybrid‐PVA, and layer‐by‐layer) had homogeneous, single‐phase fiber morphologies in which no distinct internal phase boundaries were expected based on their composition. In the core–shell sheet (Figure 4A), a distinct core–shell structure was identified in a fraction of the fibers, confirming successful coaxial electrospinning. However, many fibers exhibited a uniform internal appearance without clear phase separation, indicating that the core–shell morphology was not consistently formed across the entire fiber population. By contrast, the emulsion sheet (Figure 4B) displayed a characteristic discontinuous PCL phase embedded within the PVA/CMC‐Na matrix. The PCL domains appeared as intermittent, bead‐like inclusions along the fiber axis, and this morphology was observed in nearly all examined fibers.

**FIGURE 4:**
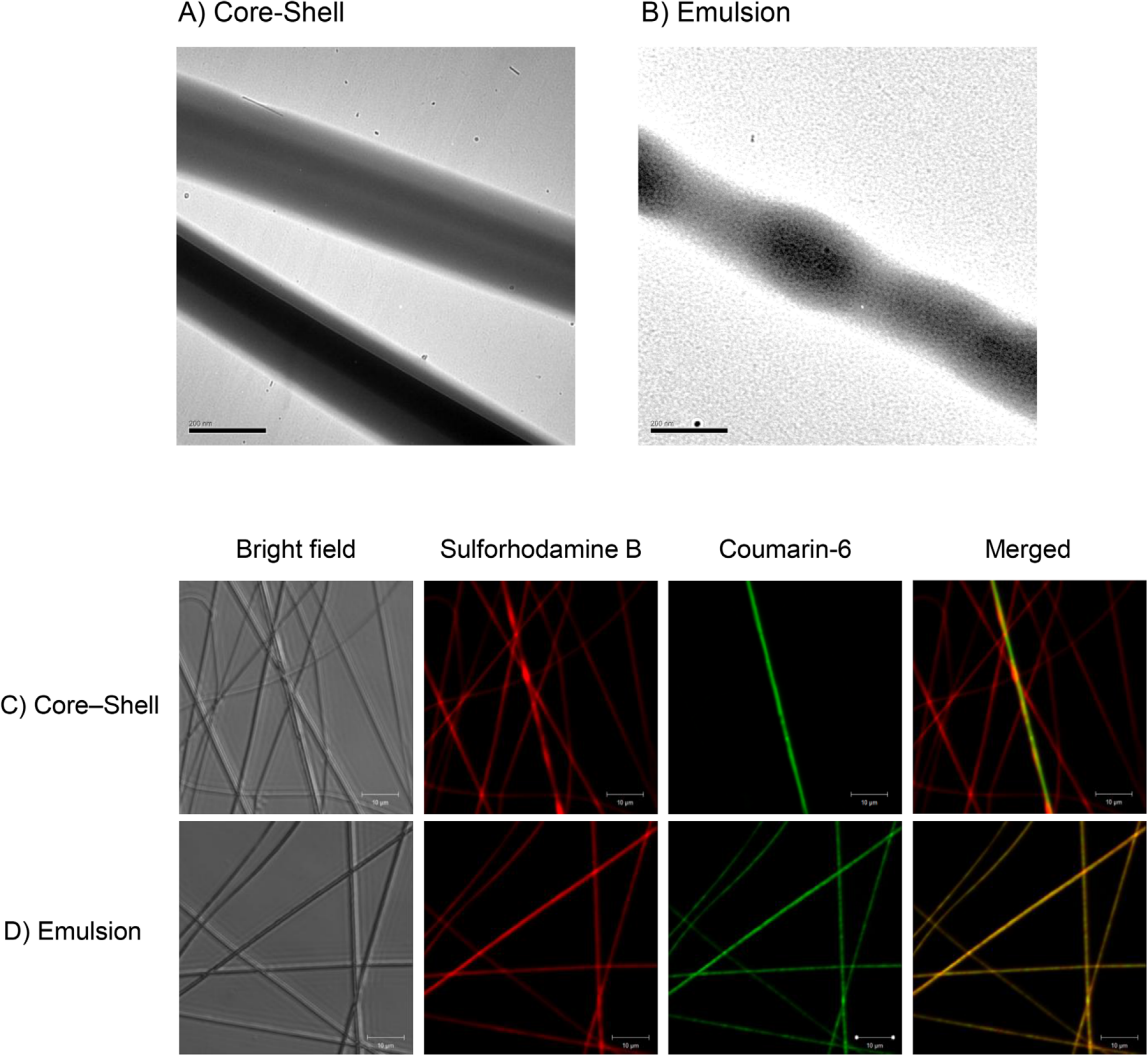
Transmission electron micrographs of (A) core–shell and (B) emulsion nanofibers. Scale bars: 200 nm. CLSM images of electrospun nanofibers incorporated with sulforhodamine B (red, PVA/CMC‐Na phase) and coumarin‐6 (green, PCL phase): (C) core–shell and (D) emulsion. Scale bars: 10 µm.

To further evaluate the spatial distribution of the two polymer phases, confocal laser scanning microscopy (CLSM) was performed using sulforhodamine B (hydrophilic fluorescent dye) incorporated into the PVA/CMC‐Na phase and coumarin‐6 (hydrophobic fluorescent dye) incorporated into the PCL phase. Representative CLSM images are shown in Figure 4C,D, and all 10 analyzed fields are provided in Figure S3. Semi‐quantitative fluorescence colocalization analysis using the Coloc 2 plugin in Fiji/ImageJ yielded thresholded Manders' coefficients (Costes automatic thresholding) of 0.55 ± 0.14 (tM1) and 0.44 ± 0.17 (tM2) for the core–shell sheet (Table S3). These results indicate partial but heterogeneous spatial colocalization of the PCL and PVA/CMC‐Na phases throughout the sheet. Since Manders' coefficients quantify fluorescence colocalization rather than the proportion of individual fibers exhibiting a true concentric core–shell structure, these values were interpreted as the degree of spatial association between the two polymer phases.

In the emulsion sheet (Figure 4D), fluorescence from both dyes was observed throughout most fibers. Coumarin‐6 fluorescence extended along the fiber axis but exhibited an intermittent, punctate distribution rather than a continuous signal, consistent with the discontinuous PCL domains observed by TEM.

### 2.4 Mechanical Properties of Nanofiber Sheets

Representative stress–strain curves obtained from tensile testing under dry and hydrated conditions are shown in Figure 5. Under dry conditions (Figure 5A), the hybrid‐PVA sheet exhibited the highest ultimate tensile strength and the greatest strain at break, indicating superior mechanical strength and flexibility (Table S4). Although incorporation of PCL reduced the tensile strength compared with the hybrid‐PVA sheet, the core–shell sheet maintained relatively high mechanical strength among the PCL‐containing nanofiber sheets (Table S4).

**FIGURE 5:**
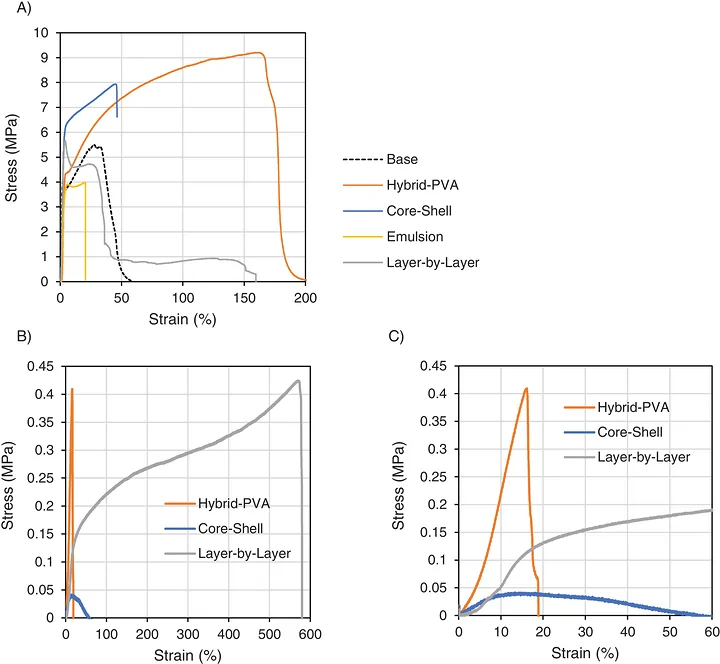
Representative stress–strain curves of electrospun nanofiber sheets under dry and hydrated conditions. (A) Representative stress–strain curves obtained under dry conditions. (B) Representative stress–strain curves obtained immediately after hydration. (C) Enlarged view of the low‐strain region of the hydrated stress–strain curves. The base and emulsion sheets were not included in the hydrated tensile tests because they fractured immediately after wetting and could not be mechanically tested.

Under hydrated conditions (Figure 5B,C), the base and emulsion sheets lost their structural integrity immediately after wetting and therefore could not be subjected to tensile testing. In contrast, the hybrid‐PVA, core–shell, and layer‐by‐layer sheets remained mechanically testable after hydration. Hydration markedly reduced the Young's modulus and ultimate tensile strength of all tested nanofiber sheets (Table S5). Among the hydrated specimens, the layer‐by‐layer sheet exhibited the highest ultimate tensile strength, whereas the core–shell sheet exhibited lower stiffness while maintaining its structural integrity throughout the tensile test (Table S5).

### 2.5 Adhesive Properties of Nanofiber Sheets

The adhesive strength of the nanofiber sheets was quantitatively evaluated against a hydrated collagen membrane (Figure 6). The base, hybrid‐PVA, core–shell, emulsion, and the PVA side of the layer‐by‐layer sheet exhibited comparable adhesive strengths, ranging from approximately 0.004 to 0.005 N mm^−2^. In contrast, the PCL side of the layer‐by‐layer sheet showed a lower adhesive strength than the other groups.

**FIGURE 6:**
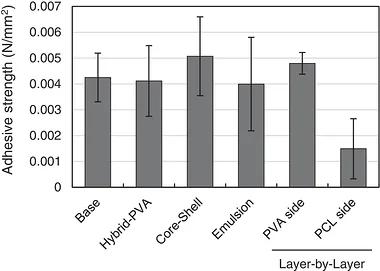
Adhesive strength of electrospun nanofiber sheets under hydrated conditions. Adhesive strength was measured against a hydrated collagen membrane. For the layer‐by‐layer sheet, both the PVA/CMC‐Na side and the PCL side were evaluated separately. Data are presented as the mean ± SD (*n* = 3).

### 2.6 Water Immersion Stability

The stability of the nanofiber sheets under aqueous conditions was evaluated by immersing the specimens in distilled water for 1 month. Figure 7A shows representative photographs of each sample before immersion, immediately after immersion, and after 1 month. The base sheet dissolved completely within the immersion period and could not be recovered for SEM analysis. By contrast, all other sheet types (hybrid‐PVA, core–shell, emulsion, and layer‐by‐layer) were intact after 1 month, indicating improved water durability. The water containing the emulsion sheet became turbid during immersion, likely due to the release of PCL particles following the dissolution of the PVA/CMC‐Na phase.

**FIGURE 7:**
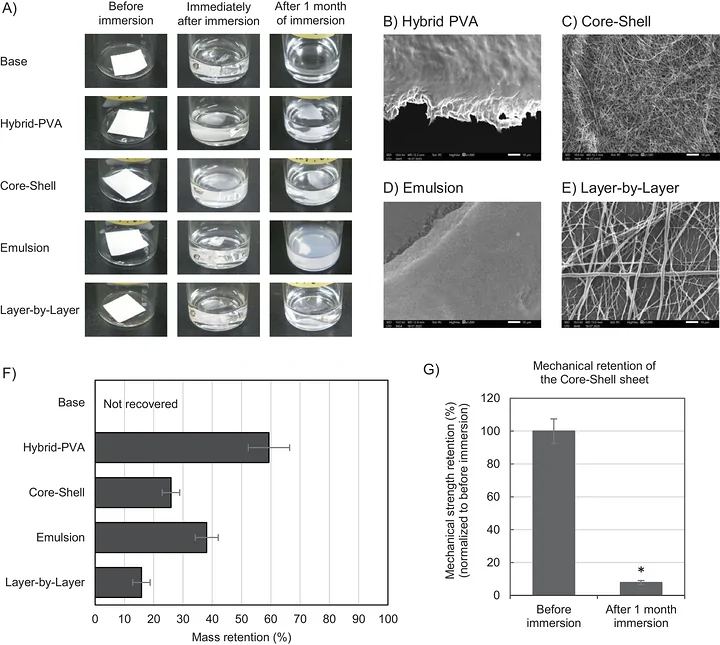
Stability and degradation behavior of the electrospun nanofiber sheets after water immersion. (A) Photographs of sheets before immersion (left), immediately after immersion in distilled water (middle), and after 1 month of immersion (right). (B–E) Scanning electron micrographs of dried sheets after 1 month of immersion: (B) hybrid‐PVA, (C) core–shell, (D) emulsion, and (E) layer‐by‐layer. Scale bars: 10 µm. (F) Residual weight (%) of the nanofiber sheets after 1 month of immersion in PBS (mean ± SD, *n* = 3). (G) Mechanical strength retention (%) of the core–shell nanofiber sheets after 1 month of immersion in PBS. The ultimate tensile strength before immersion was defined as 100%, and the value after immersion was normalized accordingly (mean ± SD, *n* = 3). *p* < 0.001 versus before immersion (Welch's unpaired *t*‐test).

SEM images of the dried sheets after 1 month of immersion are shown in Figure 7B–E. For the hybrid‐PVA (Figure 7B) and emulsion (Figure 7D) sheets, individual fibers were not distinguishable, and the material appeared as a continuous film‐like structure, suggesting fusion of the polymer phase during immersion. The core–shell sheet (Figure 7C) retained a densely packed PCL fiber network. The layer‐by‐layer sheet (Figure 7E) also retained undissolved PCL fibers. However, its network structure was looser than that of the core–shell sheet.

To quantitatively evaluate the degradation behavior under physiologically relevant conditions, additional immersion studies were performed in phosphate‐buffered saline (PBS) for 1 month. As shown in Figure 7F, the hybrid‐PVA sheet exhibited the highest residual weight (59.4% ± 7.1%), followed by the emulsion (38.1% ± 3.9%), core–shell (25.9% ± 3.0%), and layer‐by‐layer (15.8% ± 2.9%) sheets, whereas the base sheet completely dissolved. These results indicate that the structural architecture of the nanofiber sheets markedly influenced their degradation behavior.

The mechanical integrity of the core–shell sheet after PBS immersion was further evaluated by tensile testing. As shown in Figure 7G, the ultimate tensile strength after 1 month of immersion decreased significantly to 7.9% of its initial value (*p* < 0.001), indicating substantial degradation of the sheet despite retention of the PCL fibrous network.

### 2.7 Comparative Performance Assessment in an Animal Model of Pulmonary Fistula

We evaluated the sealing performance of all sheet types and an existing sealing material using a standardized pulmonary fistula model in beagle dogs (Figure 8). Artificial lung injuries were created by needle puncture. Air leak rates were then quantified using the Thopaz+ digital drainage system to standardize baseline fistula severity prior to treatment. For comparative evaluation among the different sheet types, models exhibiting an air leak volume of 40 mL/min on the monitor and maintaining a stable leak condition during pre‐treatment assessment were included. The comparative performance assessment was conducted with randomized testing order and blinded performance assessment, as described in the Experimental Section (see Sections 4.5 and 4.6 for methodological details). Fibrin glue was used only in combination with the existing product and was not applied to the nanofiber sheets. Figure 9 summarizes the scoring results for the closure performance of each sheet and an existing product consisting of a reinforcement sheet combined with fibrin glue. Compared to the existing product, the nanofiber sheets demonstrated superior performance in vertical rupture resistance and lateral leakage resistance. All sheet types, excluding the emulsion sheet, withstood a pressure loading of up to 30 cmH_2_O and achieved full scores for both parameters. By contrast, marked differences were observed across the four sheet types regarding liquid exposure tolerance. The emulsion sheet failed immediately upon exposure to pleural fluid (score: 1), whereas the hybrid‐PVA, layer‐by‐layer, and core–shell sheets exhibited seal retention scores under wet conditions of 3, 4, and 5, respectively. The final overall scores, reflecting the balance among the three performance parameters, were 52, 75, 27, and 70 for the hybrid‐PVA, core–shell, emulsion, and layer‐by‐layer sheet, respectively. The core–shell sheet exhibited the best overall performance, whereas the existing product scored 30. These results support the superior pressure resistance and adhesive performance of the nanofiber sheets and indicate that sheet structure markedly affects the ability to maintain sealing performance in vivo.

**FIGURE 8:**
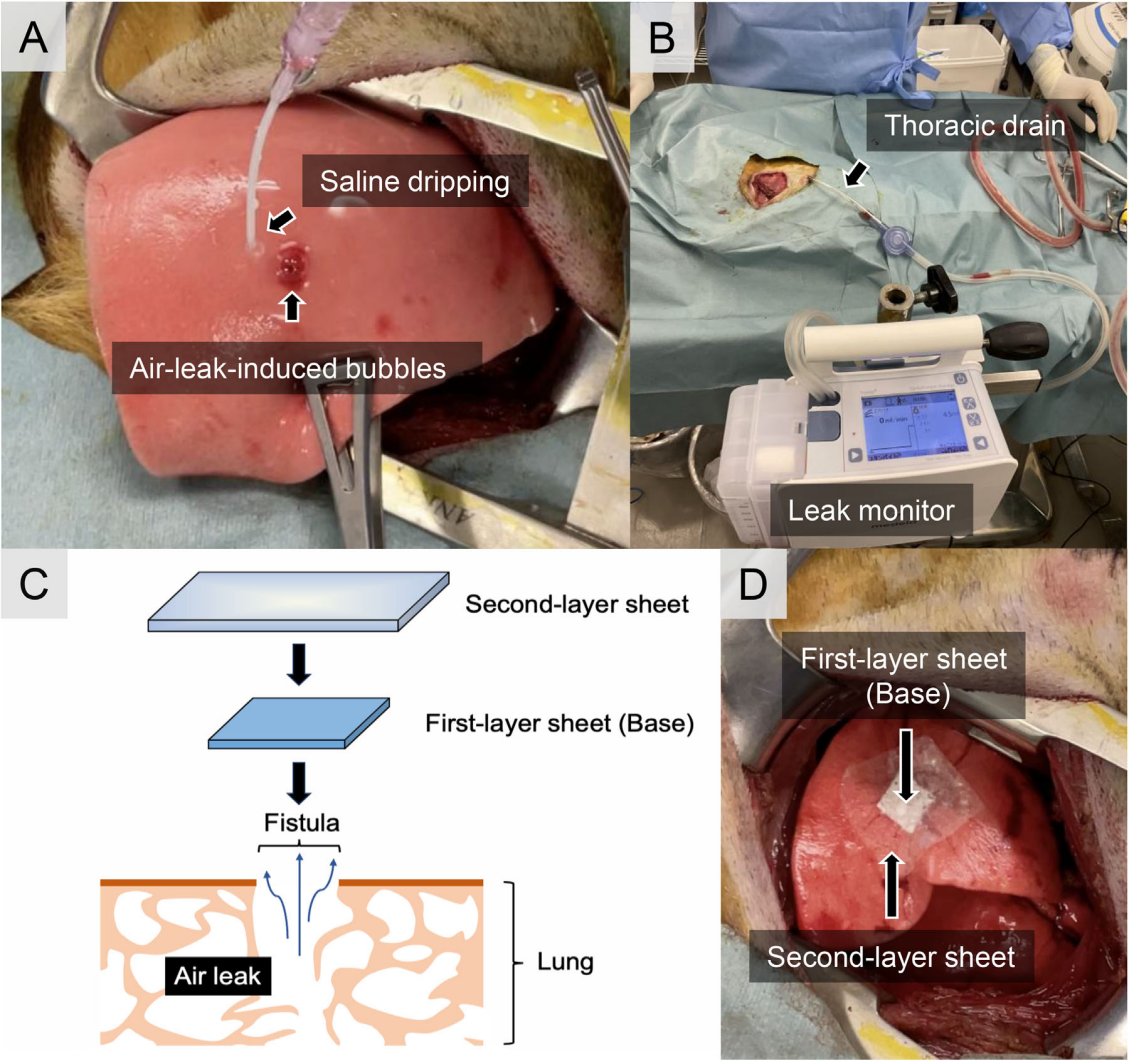
Evaluation of sheet performance using a pulmonary fistula model. (A) A standardized lung injury site. Bubbles were observed on the fistula, indicating air leakage. (B) Quantification of air leakage from the fistula using the Thopaz+ digital chest drainage system. (C) Schematic illustration of the dual‐covering therapeutic approach. A highly hydrophilic base sheet was applied as the first layer, followed by the experimental sheet as the second layer to compare their performance. (D) Appearance after the sheets were applied.

**FIGURE 9:**
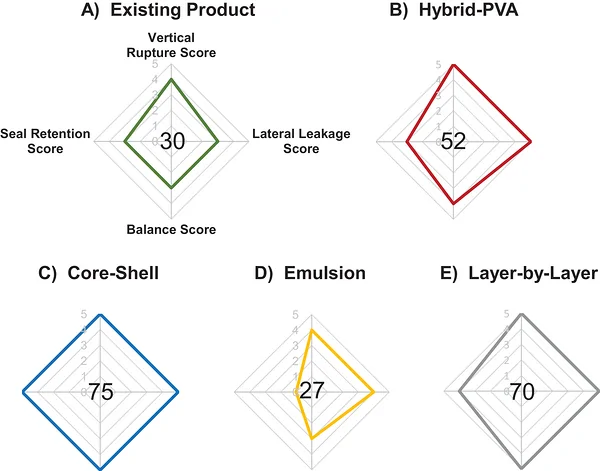
Radar chart illustrating the closure performance of the four types of nanofiber sheets and an existing product. (A) Existing product. (B) Hybrid‐PVA, (C) core–shell, (D) emulsion, and (E) layer‐by‐layer sheet. The items in the radar chart represent the vertical rupture resistance score, lateral leakage resistance score, seal retention score under wet conditions, and balance score. The value at the center of the radar chart represents the overall score. The existing product as well as the four types of nanofiber sheets were evaluated using the same assessment method with a biological pulmonary fistula model.

### 2.8 Preclinical Therapeutic Evaluation in Animal Pulmonary Fistula Model

Using the core–shell sheet, which demonstrated the best performance in comparative performance assessment, we treated the animal pulmonary fistula model. Figure 10A shows the air leak volumes measured before and after sheet application (*N* = 5). Each dot indicates a measured value of the average air leak volume during a 10‐min period between 50 and 60 min of the 1 h monitoring session conducted before and after treatment. The average air leak volume before treatment was 46.2 mL/min. By contrast, after treatment, the average air leak volume decreased to 1.8 mL/min; however, this reduction did not reach statistical significance (Wilcoxon signed‐rank test, *p* = 0.063). Variation in air leak volume was observed among individual subjects, likely reflecting inter‐individual differences. Nevertheless, the post‐treatment air leak volume was <10 mL/min in all five animals, meeting the criterion for successful closure (≤ 10 mL/min). This threshold represents a clinically meaningful value used as an indicator of the cessation of pulmonary air leakage [14, 15]. Time‐course changes in air leak volume during the 1‐h periods before and after treatment for each individual are shown in Figure S4. Air leak volumes remained low throughout the post‐treatment monitoring period in all animals. In one animal, a transient elevation in the air‐leak reading was observed during bucking (a coughing‐like response to the endotracheal tube under general anesthesia), consistent with transient expulsion of intrathoracic air rather than sustained air leakage from the sealed site.

**FIGURE 10:**
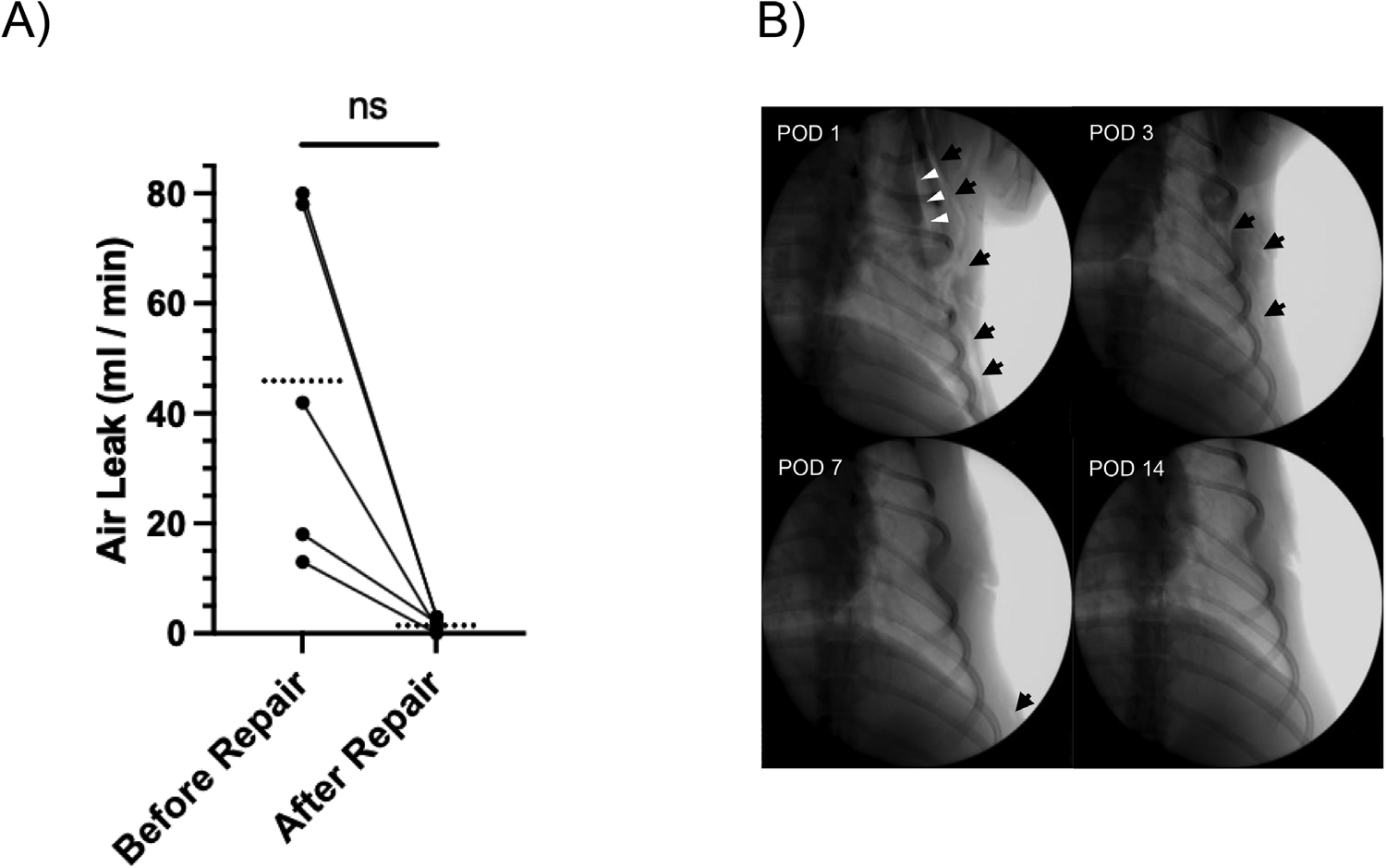
Evaluation of acute air‐leak closure performance using the nanofiber sheet. (A) Changes in leak volume before and after treatment (*N* = 5). Each dot represents the measured leak volume before or after treatment. These values correspond to the mean leak volume recorded during the 10‐min interval between 50 and 60 min of continuous monitoring. The dotted line indicates the mean leak volume of the five animals. The median (IQR) leak volume was 42 (18–78) mL/min before treatment and 2 (1–3) mL/min after treatment. Pre‐ and post‐treatment leak volumes were compared using the Wilcoxon signed‐rank test. (B) Temporal changes in chest radiographs. Arrows indicate the distribution of subcutaneous emphysema, and arrowheads indicate residual intrapleural air.

Postoperative chest x‐rays revealed intrathoracic air space and subcutaneous emphysema on POD 1. However, by POD 3, re‐expansion of the lung and a trend toward resolution of the subcutaneous emphysema were observed. On POD 7, the subcutaneous emphysema had nearly resolved, and the lung was fully re‐expanded. By POD 14, the radiographic findings had normalized (Figure 10B). A similar radiographic course was observed in all three animals during the 1 month follow‐up period. The subcutaneous emphysema observed on POD 1–3 was consistent with the typical postoperative radiographic course after thoracic surgery and was considered to reflect the gradual absorption of air trapped in the thoracic cavity during surgery. Clinical and imaging assessments revealed no progression of the subcutaneous emphysema, indicating successful repair.

Blood tests showed that CRP increased from POD 1 to POD 3 and subsequently declined, returning to near‐normal levels by POD 14. White blood cell counts increased in several animals during the early postoperative period but decreased over time. These findings are consistent with an acute postoperative inflammatory response associated with surgical stress. BUN and creatinine, markers of renal function, were within the normal range. Procalcitonin levels were within the normal range throughout the study period. No signs of acute kidney injury or infection were observed during the acute phase. AST levels increased mildly on PODs 1 and 3 and returned to the normal range by POD 7. A transient postoperative increase in AST levels was not accompanied by an increase in ALT levels, which is predominantly liver‐derived and more reflective of hepatocellular injury. *γ*‐GTP levels were within the normal range throughout the study period (Figure 11A). Given that AST is also expressed in the skeletal muscle, the isolated elevation of AST—without concurrent ALT or *γ*‐GTP elevation or renal dysfunction (as indicated by stable BUN and creatinine levels)—suggests that the increase may have been caused by muscle injury due to surgical manipulation.

**FIGURE 11:**
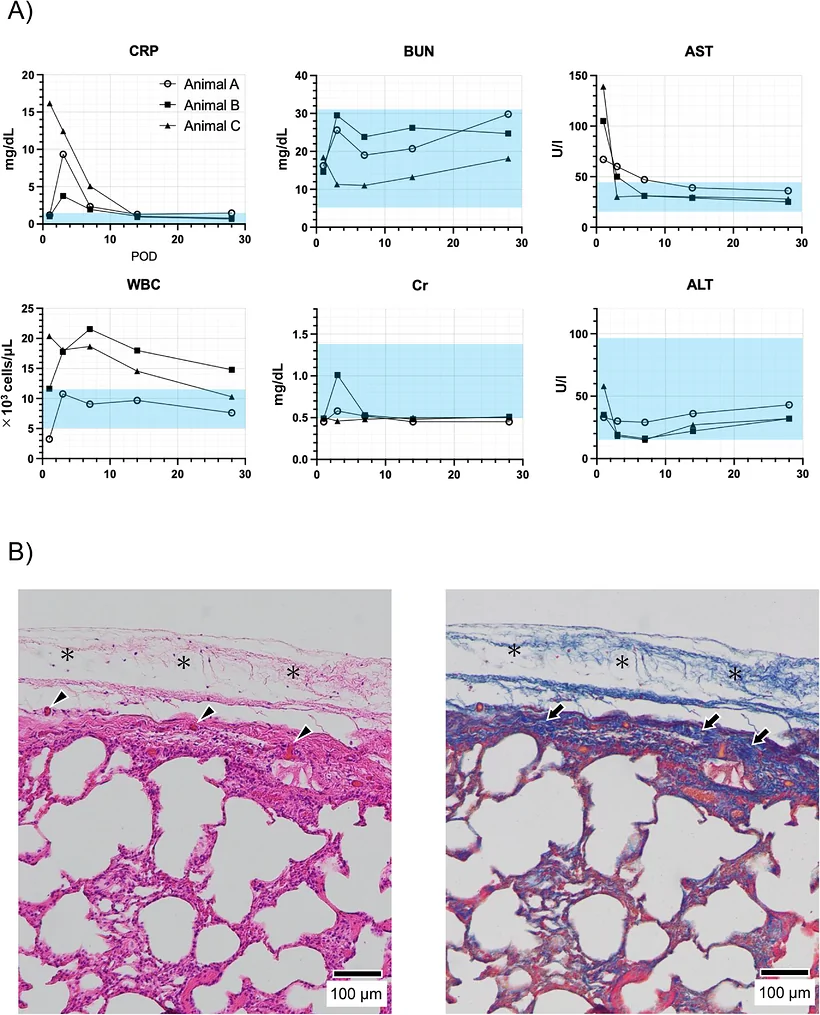
Evaluation of treatment safety using the nanofiber sheet. (A) Time‐course changes in blood test results (*N* = 3). Blue shaded areas represent normal reference ranges. (B) Histological images of the sheet application site 1 month after treatment. The left panel shows a hematoxylin and eosin‐stained histological image of a lung injury site covered with a nanofiber sheet. The right panel shows a Masson's trichrome‐stained image of the same region. Both images were obtained from the same specimen. Asterisks (*) indicate a lamellar collagenous layer containing an internal unstained area; arrowheads indicate occasional foreign body giant cells; and arrows indicate proliferating collagen fibers.

One month after surgery, histological evaluation of the lung injury site revealed fibroblast proliferation, collagen fiber deposition, and mild infiltration of inflammatory cells predominantly composed of lymphocytes. These observations are consistent with a typical wound healing process. Notably, pathological changes, such as excessive fibrosis, excessive accumulation or nodular formation of foreign body giant cells, or necrosis, were not observed at the interface between the sheet material and surrounding tissue. Occasional foreign body giant cells were present, consistent with a controlled foreign body reaction. A lamellar collagenous layer containing an internal unstained area was present along the sheet interface (Figure 11B). This unstained area possibly represented residual and/or degrading nanofiber sheet components within the collagen layer. These findings indicate that the material integrates with host tissue without inducing an excessive foreign body reaction in vivo.

## 3 Discussion

### 3.1 Study Concept

In this study, we demonstrated that the structural design of nanofiber sheets considerably influences their sealing performance as pulmonary fistula closure materials. Notably, as a tissue reinforcement material applied to the lung surface, the core–shell nanofiber sheet showed superior performance compared with other nanofiber sheet types. A comparative analysis of the in vitro and in vivo outcomes provided important insights into the key structural features required to achieve high sealing performance in the unique mechanical and physiological environment of the lung surface.

An important advancement in surgical biomaterials highlighted by this study is the departure from biologically derived products. This shift enables the resolution of intrinsic challenges inevitably associated with biological materials. To avoid reliance on fibrin network‐based adhesion mechanisms, tissue reinforcement materials designed for organ surfaces must simultaneously achieve strong adhesion and durable sealing under wet conditions. Moreover, for dynamically deforming organs, such as the lung, high flexibility is required. To satisfy these requirements concurrently, we leveraged the structural advantages of electrospun nanofiber sheets and adopted a two‐layer treatment strategy as our key conceptual approach in this study. Rather than relying on a single homogeneous material to provide adhesiveness and wet durability, this two‐layer design assigns distinct functions to each layer: the first layer (base sheet) enhances initial tissue adhesion, whereas the second layer reinforces seal retention and mechanical stability under wet conditions.

The material composition was selected according to these functional requirements. The hydrophilic polymers, PVA and CMC‐Na, provide bioadhesiveness through hydrogen bonding with polar functional groups on the tissue surface, thereby contributing to effective sealing of the injured area [16, 17]. Furthermore, the hydrophobic polymer PCL was incorporated to enhance the structural stability and pressure resistance of the sheet under wet conditions [18]. These synthetic polymers were selected based on substantial evidence of their biocompatibility [19, 20, 21, 22].

### 3.2 Comparative Discussion

As the first layer, a blended base sheet comprising PVA and CMC‐Na was used to provide tissue adhesion and effective sealing through gelation. Although this sheet exhibited sufficient adhesiveness to lung tissue, it readily gelled and dissolved even in the presence of small amounts of moisture, rendering it insufficient as a standalone sealing material (Figure 7A). Although all four second‐layer sheets exhibited similar mean fiber diameters (400–700 nm), their sealing performances differed substantially. The high specific surface area of these nanofiber sheets is expected to increase tissue contact, thereby promoting adhesion through hydrogen bonding and other intermolecular interactions while also facilitating physical interlocking with microscopic irregularities on the tissue surface [23, 24, 25, 26]. Consistent with these characteristics, quantitative adhesion testing demonstrated comparable adhesive strengths among the hydrophilic surfaces of the base, hybrid‐PVA, core–shell, emulsion, and the PVA side of the layer‐by‐layer sheet, whereas exposure of the hydrophobic PCL surface markedly reduced adhesion (Figure 6). These findings indicate that the hydrophilic PVA/CMC‐Na surface primarily governs initial adhesion to moist tissue and that differences in initial adhesive strength alone cannot account for the differences observed in sealing performance.

Among the four second‐layer designs, the hybrid‐PVA sheet was developed by incorporating fully saponified PVA (NH‐26) to strengthen intermolecular interactions between PVA chains and thereby reduce water solubility [27]. Consistent with this design concept, the dissolution rate decreased, and tensile testing demonstrated the highest tensile strength and elongation among the evaluated sheets, indicating a favorable balance between flexibility and mechanical durability. However, gradual dissolution and loss of fibrous morphology during water immersion were accompanied by limited long‐term sealing performance in vivo, suggesting that improved bulk mechanical properties alone are insufficient to ensure durable pulmonary sealing.

The emulsion sheet incorporated PCL as dispersed particles within the fiber structure. However, turbidity developed during water immersion owing to the release of PCL particles, indicating structural instability under hydrated conditions. This observation was consistent with its relatively poor sealing performance in vivo despite the presence of a hydrophobic reinforcing component.

The layer‐by‐layer sheet combined a tissue‐contacting PVA/CMC‐Na layer with an overlying PCL reinforcing layer. This design provided strong initial adhesion together with satisfactory resistance to air leakage. Nevertheless, its sealing performance under wet conditions remained inferior to that of the core–shell sheet. Although the layer‐by‐layer sheet exhibited the highest toughness under hydrated conditions (Table S5), this mechanical advantage alone did not translate into superior seal retention. One possible explanation is the structural vulnerability associated with interlayer separation, although this mechanism was not directly evaluated in the present study.

In contrast, the core–shell sheet maintained its fibrous morphology throughout 1 month of water immersion while demonstrating the highest seal retention in vivo. Since the quantitative adhesion test demonstrated comparable initial adhesive strength among the PVA‐containing sheets, the superior sealing performance of the core–shell sheet is unlikely to be explained solely by stronger adhesion. Instead, preservation of structural integrity under hydrated conditions appears to be a key determinant of durable pulmonary sealing.

### 3.3 Mechanistic Interpretation

The superior sealing performance of the core–shell sheet is likely attributable to the combined effects of hydration‐responsive mechanical behavior, preservation of fibrous architecture, and gradual biodegradation rather than to a single dominant mechanism.

Hydrated tensile testing demonstrated that the core–shell sheet maintained its structural integrity throughout mechanical loading, whereas the base and emulsion sheets rapidly lost their structural integrity immediately after wetting and could not be mechanically evaluated (Table S5). Although hydration markedly reduced the Young's modulus and ultimate tensile strength of the core–shell sheet, the fibrous network remained intact while becoming mechanically more compliant. This hydration‐induced reduction in stiffness may improve mechanical compatibility with the native lung surface without compromising structural integrity.

Mechanical compatibility between pulmonary sealants and the native visceral pleura has recently been recognized as an important factor for minimizing interfacial stress concentration, delamination, and tissue damage during repetitive respiratory deformation [28]. Although the present uniaxial tensile measurements cannot be directly compared with previously reported biaxial mechanical properties of the visceral pleura, the observed reduction in stiffness after hydration is consistent with the possibility that the core–shell sheet more closely adapts to physiological deformation while maintaining seal integrity.

The structural configuration of the core–shell sheet may also contribute to this behavior. Continuous integration of hydrophilic and hydrophobic polymer phases within individual fibers may reduce structural discontinuity during hydration. However, the contribution of this structural configuration to seal durability was not directly quantified in the present study and therefore remains a mechanistic interpretation. Fluorescence colocalization and TEM analyses demonstrated that this continuous integration was heterogeneous rather than perfectly uniform throughout the sheet. Thresholded Manders’ coefficients indicated partial spatial association between the PCL and PVA/CMC‐Na phases (Table S3), suggesting that individual fibers differed in the extent of polymer integration. Importantly, despite this structural heterogeneity, the core–shell sheet exhibited the highest seal retention in vivo. These findings suggest that complete and perfectly uniform core–shell formation in every individual fiber may not be required. Instead, localized continuous integration of hydrophilic adhesive and hydrophobic reinforcing polymer domains may contribute to preservation of structural integrity under hydrated conditions. Nevertheless, direct quantitative correlation between local fiber architecture and macroscopic sealing performance remains to be established.

The degradation study provides additional evidence supporting the potential suitability of this structural design for pulmonary applications. Although the core–shell sheet retained its fibrous morphology after 1 month of immersion in PBS, its residual weight decreased to approximately 26%, and its tensile strength decreased to less than 10% of the initial value (Figure 7F,G). These findings indicate that preservation of fibrous morphology does not necessarily reflect preservation of mechanical function. Rather, gradual dissolution of the hydrophilic shell together with progressive weakening of the fibrous network appears to reduce the mechanical contribution of the material and may help avoid long‐term persistence as a foreign body. Considering that pulmonary air leaks generally require sealing only during the early postoperative healing period, this degradation profile appears compatible with the intended clinical application. Furthermore, the limited foreign‐body giant cell response observed 1 month after implantation supports the favorable biocompatibility of this degradation process.

### 3.4 Clinical Implications

The therapeutic potential of the core–shell sheet was further supported by evaluation in a canine pulmonary fistula model, which reproduces the dynamic mechanical environment encountered in clinical thoracic surgery. When two‐layer treatment using the core–shell sheet was applied to the pulmonary fistula animal model, closure was achieved in all five cases regardless of interindividual variability in initial leak volume. The air leak volume markedly decreased throughout the measurement period (Figure S4), and no signs of recurrent leakage were observed on chest radiographs (Figure 10B), providing preliminary evidence of sustained leak control by the sheet. Although common postoperative complications following lung surgery include atelectasis, infections (bronchitis and pneumonia), pneumothorax, and hepatic or renal dysfunction [29, 30, 31], only a mild elevation of AST levels (Grade 1 according to CTCAE v5.0), presumably attributable to muscle injury associated with thoracotomy, was observed in this study. No other complications were detected. One month after treatment, histological examination revealed limited foreign body giant cell formation, consistent with a typical response to biodegradable materials. These findings support the potential of the core–shell sheet developed in this study as a pulmonary fistula closure material with favorable sealing performance and safety. Notably, this study did not employ any biologically derived materials commonly used in conventional treatments; only synthetic polymers with established in vivo safety profiles were used as the nanofiber substrate. The absence of biologically derived components may offer an additional advantage over conventional materials, further supporting the potential of the core–shell sheet as an alternative material for pulmonary air‐leak closure.

### 3.5 Limitations

This study has several limitations. First, since this study was designed as an exploratory proof‐of‐concept investigation, the number of animals used to evaluate in vivo therapeutic efficacy was limited. Therefore, although marked reductions in air leakage were consistently observed in all treated animals, larger studies will be required to confirm reproducibility and provide sufficiently powered statistical evaluation of treatment efficacy. Second, although short‐term safety and histological responses were assessed for up to 1 month, longer‐term degradation kinetics and chronic tissue responses remain to be fully characterized. Third, although fluorescence colocalization analysis demonstrated heterogeneous spatial association between the two polymer phases, the relationship between local fiber architecture and the resulting mechanical or adhesive performance remains to be established. In addition, although quantitative adhesion testing against a hydrated collagen membrane demonstrated comparable tissue adhesive strengths among the hydrophilic surfaces of the nanofiber sheets (Figure 6), the interlayer bonding strength between the PVA/CMC‐Na adhesive layer and the PCL reinforcing layer in the layer‐by‐layer sheet was not directly measured. Direct evaluation of this interlayer bonding strength would help clarify the contribution of interlayer delamination to sealing performance and further improve the mechanistic understanding of the structural advantages of the core–shell architecture. Fourth, although the sealing performance of the core–shell sheet was successfully demonstrated in a dynamic in vivo pulmonary model, dedicated cyclic mechanical testing under controlled hydrated conditions was not performed. Future studies should evaluate the fatigue resistance and long‐term mechanical durability of the core–shell sheet under physiologically relevant cyclic loading conditions. Finally, although hydrated tensile properties were evaluated in this study, direct mechanical comparison with native lung tissue was not performed because of the technical challenges associated with obtaining reproducible tensile properties from highly compliant pulmonary tissue.

### 3.6 Future Perspectives

In this study, only four types of sheets were evaluated. Further investigation is required to determine the optimal configuration for application to the lung surface. Nevertheless, the core–shell nanofiber sheet, fabricated by leveraging the unique advantages of the electrospinning method, exhibited favorable adhesiveness and sealing retention in vivo, suggesting its potential as a sealing material for pulmonary air leak closure. In future research, its applicability can be expanded to other organs. Incorporation of anti‐inflammatory drugs or growth factors into nanofibers may enable the development of multifunctional tissue‐reinforcing materials with drug delivery capabilities.

## 4 Experimental Section

### 4.1 Materials

PVA was provided by Mitsubishi Chemical (Tokyo, Japan). Two grades were used in this study: Gohsenol EG‐40P (degree of hydrolysis: 86.5–89.0 mol% and viscosity: 36.6–49.4 mPa·s [4% aqueous solution]) and NH‐26 (degree of hydrolysis: ≥99.0 mol% and viscosity: 60–70 mPa·s [4% aqueous solution]). Sodium carboxymethyl cellulose (CMC‐Na) was purchased from FUJIFILM Wako Pure Chemical (Osaka, Japan). Poly(ε‐caprolactone) (PCL, average Mn ∼80 000) was purchased from Sigma–Aldrich (St. Louis, MO, USA). Coumarin‐6 (laser grade) and sulforhodamine B were purchased from MP Biomedicals (Santa Ana, CA, USA). Neoveil Sheet was purchased from Gunze Medical Corporation (Kyoto, Japan). Beriplast P Combi‐Set Tissue Adhesive was purchased from CSL Behring (Tokyo, Japan) and used as an existing fibrin‐based surgical sealant. Ethanol, ethyl acetate, *N*,*N*‐dimethylformamide (DMF), and chloroform were used as special reagent grade solvents.

### 4.2 Electrospinning‐Based Fabrication of Nanofiber Sheets

#### 4.2.1 General Procedure

One type of first‐layer sheet and four types of second‐layer sheets were fabricated using a laboratory‐scale electrospinning apparatus (Fluidnatek LE‐50; Bioinicia, Spain), equipped with a high‐voltage power supply and a static plate collector. All fibers were collected as randomly oriented nonwoven mats. Electrospinning was performed under controlled environmental conditions: temperature 20°C–25°C and relative humidity 40%–50%. Each sheet type was fabricated using polymer solutions specifically formulated to achieve the desired fiber morphology and physical properties. The composition of each electrospinning solution is summarized in Table 1, and the electrospinning parameters are detailed in Table 2.

After electrospinning was complete, all sheets were cut to predetermined sizes before use. Base sheets were trimmed into 1.5 cm × 1.5 cm squares and applied as the first‐layer sealing sheet, whereas all second‐layer sheets (hybrid‐PVA, core–shell, layer‐by‐layer, and emulsion) were trimmed into 2.5 × 2.5 cm squares and placed over the base sheet.

#### 4.2.2 First‐Layer Sheet

The first layer was designed to form a gel under wet conditions, exhibit bioadhesive properties, and provide initial closure of the fistula. The first‐layer sheet, referred to as the base sheet, was a 1.5 × 1.5 cm square composed of PVA and CMC‐Na. Both PVA and CMC‐Na are hydrophilic polymers that swell and gel upon exposure to small amounts of water and have documented mucoadhesive and tissue‐adhesive properties [32, 33, 34, 35]. Both are components of approved pharmaceutical products and medical devices, such as ophthalmic formulations and the Seprafilm adhesion barrier, underscoring their biocompatibility and clinical applicability [20]. The base sheet was prepared by electrospinning an aqueous polymer solution containing 9.58% partially hydrolyzed PVA EG‐40P and 1.06% CMC‐Na through a single 16G stainless‐steel needle.

#### 4.2.3 Second‐Layer Sheet

The second layer was intended to reinforce the airtight initial seal achieved by the first layer and enhance seal retention under wet conditions. To identify a suitable structure for this second layer, we designed and evaluated four types of nanofiber sheets. One of these sheets was prepared by incorporating fully hydrolyzed PVA into the fiber matrix to enhance water resistance. Fully hydrolyzed PVA exhibits water resistance due to its high crystallinity and strong intermolecular hydrogen bonding arising from densely distributed hydroxyl groups. This sheet was referred to as the hybrid‐PVA sheet. The remaining three types—core–shell, emulsion, and layer‐by‐layer sheets—were designed by integrating hydrophobic and biodegradable polymer PCL into the fiber structure using different fabrication techniques. PCL is a hydrophobic, biodegradable polyester with established biocompatibility and is widely used in approved medical devices, including absorbable surgical sutures, nerve guides, and long‐acting drug delivery implants [21, 22]. By comparing these four designs, we identified the most promising formulation for sealing pulmonary fistulas under clinically relevant conditions.

#### 4.2.4 Hybrid‐PVA Sheet

The hybrid‐PVA sheet was prepared by electrospinning an aqueous polymer solution containing 1.33% partially hydrolyzed PVA (EG‐40P) and 6.67% fully hydrolyzed PVA (NH‐26) through a single 16G stainless‐steel needle [36].

#### 4.2.5 Core–Shell Sheet

The core–shell sheet was prepared by electrospinning two polymer solutions using a coaxial spinneret with an inner needle diameter of 0.6 mm and an outer needle diameter of 1.4 mm. The shell solution contained 9.58% PVA (EG‐40P) and 1.06% CMC‐Na in water, and the core solution contained 10.71% PCL in chloroform/DMF (8:2, *v/v*). Each solution was delivered independently from separate syringes to form a continuous core–shell fiber structure.

#### 4.2.6 Emulsion Sheet

The emulsion was prepared by mixing an aqueous solution containing 7.8% *w/w* PVA (EG‐40P) and 0.87% *w/w* CMC‐Na with an organic solution containing 12% *w/w* PCL in ethyl acetate at a 6.2/4.0 (*w/w*) ratio. The final concentrations of PVA, CMC‐Na, and PCL in the mixed solution are given in Table 1. The mixture was premixed manually for 30 s, homogenized at 8000 rpm for 5 min, allowed to rest for 3 min, and homogenized for 5 min using a rotor–stator homogenizer (Polytron Model PT10‐35, Kinematica AG, Luzernerstrasse, Switzerland) to yield a stable emulsion [37]. The emulsion was electrospun through a single 16G stainless steel needle. The emulsion was electrospun immediately after preparation. No visible phase separation or sedimentation was observed during the electrospinning process.

#### 4.2.7 Layer‐by‐Layer Sheet

The layer‐by‐layer sheet was prepared in two sequential steps using a single 16G stainless steel needle. The first‐spun layer consisted of 10.71% PCL in chloroform/DMF (8:2, *v/v*). Immediately thereafter, the second‐spun layer was electrospun directly onto the first‐spun layer from an aqueous solution containing 9.58% PVA (EG‐40P) and 1.06% CMC‐Na. No intermediate drying or additional bonding treatment was applied between the two electrospinning steps.

### 4.3 Physicochemical Characterization of the Nanofiber Sheets

The morphology of the electrospun nanofiber sheets was examined using scanning electron microscopy (SEM; JCM‐7000 NeoScope, JEOL, Tokyo, Japan). Fiber diameters and the area fractions of fibers and pores were quantified from SEM images acquired at ×2000 magnification using MIPAR software (version 4.3.0; MIPAR Image Analysis, Columbus, OH, USA) with a modified fiber analysis recipe optimized for each image. One SEM image was analyzed for each sheet type or surface, and all fibers detected within the field of view were included in the automated analysis. Images acquired at ×2000 magnification (Figures S1 and S2) were selected to include a larger number of fibers within the field of view than the ×5000 images presented in Figure 3. For core–shell and emulsion nanofibers, TEM (JEM2100FGK, JEOL, Tokyo, Japan) was used to observe the internal structure of specimens. Additionally, these two sheet types were prepared with sulforhodamine B incorporated into the PVA/CMC‐Na phase and coumarin‐6 incorporated into the PCL phase and imaged using a confocal laser scanning microscope (LSM‐700, Carl Zeiss, Jena, Germany) to visualize the spatial distribution of each component.

The tensile properties of the nanofiber sheets were evaluated using a universal testing machine (EZ‐SX, Shimadzu, Kyoto, Japan) equipped with a 50‐N load cell. Rectangular specimens (5 mm × 30 mm) were prepared using a pneumatic punch cutter. The width and thickness of a specimen were measured using a Mitutoyo micrometer to calculate the cross‐sectional area. Each strip was mounted with a gauge length of 17 mm, and tests were performed at 25°C ± 2°C and 40% ± 5% relative humidity with a crosshead speed of 0.5 mm/min. For hydrated tensile testing, the specimen was initially mounted with a gauge length of 18 mm and then adjusted to 17 mm to introduce a slight slack, compensating for specimen shrinkage upon hydration. Subsequently, 0.5 mL of distilled water was gently applied to each side of the specimen using a micropipette (total volume: 1.0 mL), and tensile testing was initiated immediately after wetting.

The adhesive properties of the nanofiber sheets were evaluated using a universal testing machine (EZ‐SX, Shimadzu, Kyoto, Japan). A collagen membrane (CLF‐01, Koken Co., Ltd., Tokyo, Japan) was cut into 30 × 30 mm squares and fixed onto a 10.7% (*w/w*) gelatin gel using a cyanoacrylate adhesive. The prepared substrate was allowed to stand for 30 min before testing. Circular nanofiber specimens with a diameter of 15 mm were attached to the upper fixture using double‐sided adhesive tape. The collagen membrane was wetted with distilled water for 1 min, after which excess water was removed, and the collagen membrane–gelatin substrate was fixed to the lower fixture. The upper fixture was lowered at a crosshead speed of 0.1 mm s^−1^ until a compressive load of 1 N was reached. The compressive load was maintained for 20 s, after which the upper fixture was withdrawn at the same speed of 0.1 mm s^−1^. The maximum force recorded during detachment was divided by the contact area of the circular specimen and expressed as adhesive strength (N mm^−2^).

A water immersion stability test was conducted to assess the morphological changes of the nanofiber sheets under prolonged aqueous exposure. Each 1.5 cm × 1.5 cm sheet was placed in a glass vial containing 3 mL of distilled water, sealed, and stored at 40°C in the dark for 1 month. The specimens were visually inspected, and their surface morphology was examined by SEM. The residual weight was measured to determine the mass retention of each sheet. In addition, the mechanical strength retention of the core–shell nanofiber sheets was evaluated by tensile testing after drying. The ultimate tensile strength after immersion was normalized to that before immersion, which was defined as 100%.

### 4.4 Ethics Review

All animal procedures were approved by the Animal Ethics Committee of Aichi Medical University (approval No. 2024‐10). This approval covered the animals used in both the pilot and main studies described in the present study. All experiments were conducted in accordance with the ARRIVE guidelines, the National Research Council's Guide for the Care and Use of Laboratory Animals, and the principles of the 3Rs.

### 4.5 Experimental Animals and Surgical Procedure

Beagle dogs (9 months old, ∼10 kg in body weight; KITAYAMA LABES Co., Ltd., Nagano, Japan) were used as lung fistula models. General anesthesia was administered subcutaneously using a combination of three agents: medetomidine (0.05 mg/kg; Meiji Seika Pharma Co., Ltd., Tokyo, Japan), butorphanol (0.02 mg/kg; Meiji Seika Pharma Co., Ltd., Tokyo, Japan), and midazolam (0.2 mg/kg; Maruishi Pharmaceutical Co., Ltd., Osaka, Japan) and maintained with total intravenous anesthesia using propofol (6–18 mL/h; Maruishi Pharmaceutical Co., Ltd., Osaka, Japan) and remifentanil (0.2–0.4 µg/kg/min; Janssen Pharmaceutical K.K., Tokyo, Japan). Thoracotomies were performed through 10 cm skin incisions at the fourth intercostal space. Artificial lung injuries were created by puncturing the lung surface with 18‐gauge needles. Air leakage was confirmed by observing bubble formation after saline was dripped onto the injury sites while the ventilator airway pressure was maintained at 15 cmH_2_O (leak test). Air leakage was quantified by inserting 14‐Fr thoracic catheters through the sixth intercostal space, positioning the catheter tips near the lung apex, and connecting them to a digital low‐pressure suction device (Thopaz+, Medela AG, Switzerland). Air leak flow rates, averaged over 10 min intervals, were exported for analysis.

To quantify the severity of air leakage in pulmonary fistula animal models, air leakage was measured for 1 h under pressure‐controlled ventilation at an airway pressure of 30 cmH_2_O using the Thopaz+ digital drainage system. For comparative performance evaluation among the different sheet types, only animals exhibiting an air leak volume of 40 mL/min on the Thopaz+ monitor and showing a stable leak condition without fluctuation in the displayed leak volume during the pre‐treatment assessment period were included. The testing order of the different sheet types was randomly determined by an investigator who prepared the test sheets but was not involved in the surgical procedures or performance assessments. The prepared sheets were then provided to a separate operator, who was blinded to the identity, structural characteristics, and fabrication details of the nanofiber sheets. The pulmonary fistula models were subsequently treated using a first‐layer sheet and a second‐layer sheet, and the same operator performed the surgical procedures and performance assessments according to the predefined evaluation protocol described below.

In the treatment trial, air leakage was quantified for 1 h prior to intervention, after which the core–shell sheets were applied to close the fistulas (total *N* = 5; pilot study, *N* = 2; main study, *N* = 3). All animals that underwent the core–shell therapeutic evaluation were included in the analysis, and no animals or available acute air‐leak data were excluded. The operator who performed the therapeutic evaluation was aware that the core–shell sheet was being used for treatment and was therefore not blinded to the treatment. Posttreatment air leakage was evaluated for 60 min under pressure‐controlled ventilation at 30 cmH_2_O with a respiratory rate of 15 breaths/min. Treatment was considered successful when air leak was ≤ 10 mL/min, a threshold regarded as the clinical cessation of pulmonary air leakage. In the pilot study, the animals were euthanized immediately after leak assessment according to the experimental protocol, whereas in the main study, the animals were followed for 1 month postoperatively to evaluate the long‐term stability of fistula closure and in vivo safety.

### 4.6 Scoring System for the In Vivo Evaluation of Sheet Performance

To function effectively as a reinforcement material for lung injuries, a sealant must provide sufficient pressure resistance and strong tissue adhesion. Previous studies have shown that pulmonary sealants can fail either by perforation under airway pressure or by detachment from the lung surface, resulting in air leakage [8, 9, 10, 38]. Furthermore, the lung surface remains continuously moist and undergoes repetitive deformation during respiration, making the ability to maintain a seal under wet conditions and repetitive respiratory loading essential. Based on these considerations, three parameters—vertical rupture resistance, lateral leakage resistance, and seal retention under wet conditions—were established as the core components of the scoring system. Each parameter was evaluated using a five‐point scale (1–5 points), with higher scores indicating superior performance. The scoring thresholds were based on the stepwise pressure‐loading protocol used in this study, which covered the range of airway pressures commonly employed during intraoperative air‐leak testing in thoracic surgery, as summarized in Table S1.

The scores for vertical rupture resistance and lateral leakage resistance were determined based on the maximum airway pressure at which the sealing sheet failed. Airway pressure was set at 30 cmH_2_O as the upper limit for pressure loading. This corresponds to the typical maximum pressure used during fistula closure tests in lung surgery, considering the risk of airway mucosal injury [39, 40, 41]. Specifically, after repairing the damaged area with the sheet, the airway pressure of the ventilator gradually increased from 15 to 30 cmH_2_O in 5 cmH_2_O increments (see Video S1). The pressure at which a hole formed in the sheet over the fistula or at which the sheet lifted and side leakage occurred was recorded and used for scoring. In cases where the sheet was lifted and lateral leakage occurred, the sheet was secured using ring‐shaped surgical forceps, and its vertical rupture resistance was investigated. Sheets that exhibited immediate perforation over the fistula after application were considered unsuitable for evaluation because subsequent assessment of pressure resistance, lateral leakage resistance, and seal retention could not be performed. Therefore, such sheets were predefined as an exclusion criterion and were excluded from scoring analysis. However, no sheet met this exclusion criterion in the present study.

The seal retention score under wet conditions was determined based on the duration for which the applied sheet maintained closure after saline was sprayed onto the sheet surface to ensure sufficient wetting of the entire sheet. During this evaluation, airway pressure was maintained at 20 cmH_2_O while repetitive respiratory loading was continuously applied by mechanical ventilation (see Video S2).

As an auxiliary metric for comparing sheet performance, a balance score was defined. If sheet performance were evaluated solely based on the sum of the three individual scores, a sheet with one markedly deficient property could still receive a relatively high overall evaluation. Such an outcome could result in a discrepancy between the scoring results and the practical performance required of a clinically useful sealing sheet. Since failure in any single performance domain may compromise the practical utility of a pulmonary sealant, the maximum balance score was limited to 3 whenever any of the three performance metrics received a score of ≤ 2. Sheet performance was ultimately compared using an overall score, calculated as the sum of the three individual scores multiplied by the balance score (Table S1).

### 4.7 Postoperative Evaluation

Postoperative evaluations were performed to assess the seal integrity of the sheet affixed to the lung, potential hematologic toxicity associated with the sheet, and other related complications (*N* = 3). In addition to recording clinical signs, chest radiographs were obtained on PODs 1, 3, 7, and 14. To monitor inflammatory responses, liver and kidney dysfunction, and signs of infection, blood samples were collected on PODs 1, 3, 7, 14, and 28 to measure white blood cell count as well as CRP, AST, ALT, BUN, creatinine, *γ*‐GTP, and procalcitonin levels. One month after surgery, the animals were humanely euthanized following institutional ethical guidelines. Lung tissue samples were promptly harvested; fixed for 48 h in 10% neutral‐buffered formalin; processed; embedded using an enclosed automated tissue embedding system; sectioned at 5‐µm thickness; and stained with hematoxylin and eosin and Masson's trichrome for histological analysis. Histological assessment was not performed under blinded conditions, as the evaluator was aware that the specimens were obtained from lungs treated with the core–shell sheet.

### 4.8 Statistical Analysis

Ultimate tensile strength values of the core–shell sheets before and after 1 month of immersion in PBS were compared using Welch's unpaired *t*‐test. Air leak volumes before and after treatment were compared using the Wilcoxon signed‐rank test because of the small sample size (*N* = 5). All statistical analyses were two‐sided, and *p*‐values < 0.05 were considered statistically significant.

## Author Contributions

**Tomohito Okubo**: conceptualization, methodology, investigation, formal analysis, visualization, writing – original draft, project administration, funding acquisition. **Kouji Hara**: conceptualization, methodology, formal analysis, resources. **Naoyuki Hatayama**: investigation, validation, data curation, visualization. **Kota Takahashi**: investigation, validation. **Eriko Yamazoe**: investigation, data curation, visualization, writing – review and editing. **Takaaki Ito**: validation, writing – review and editing. **Takao Takeuchi**: investigation, data curation, formal analysis. **Yoko Koide**: investigation, data curation, formal analysis. **Mayumi Yamada**: investigation, data curation, formal analysis. **Munekazu Naito**: formal analysis, resources, supervision. **Kohei Tahara**: conceptualization, methodology, investigation, formal analysis, visualization, writing – review and editing, supervision, funding acquisition.

## Funding

This work was supported by the Translational Research Program, Seeds A (2024), Fujita Base, and the Japan Agency for Medical Research and Development under the Medical Device R&D Promotion Program–Challenge Type (2025).

## Conflicts of Interest

All authors have reviewed the journal's policy on the disclosure of potential conflicts of interest. K.H. is an employee of Mitsubishi Chemical Corporation, which provided the PVA used in this study. T.O., K.H., M.N., and K.T. are co‐applicants on a pending Japanese patent application (Application No. 2025–037776) related to the nanofiber sheet for the treatment of injured tissue, including its fabrication method and therapeutic application. At present, there are no commercialization, licensing, or royalty agreements related to this technology. The above relationships and interests are disclosed as potential competing interests. All other authors declare no competing interests.

## Supporting information




**Supporting File 1**: adhm71789‐sup‐0001‐SuppMat.docx.

**Supporting File 2**: adhm71789‐sup‐0002‐VideoS1.mp4.

**Supporting File 3**: adhm71789‐sup‐0003‐VideoS2.mp4.

## Data Availability

The datasets used and analyzed during the current study are available from the corresponding author on reasonable request.
