# Supplementary material for: Electrospun Core–Shell Bioadhesive Nanofiber Sheets Enable Durable Pulmonary Air Leak Sealing Under Wet and Dynamic Conditions
Source: Adv Healthc Mater. 2026 Oct 8;15(40):e71789. doi: 10.1002/adhm.71789 (PMC13712213; doi:10.1002/adhm.71789)
Supplement: Supplementary file 1 [file ADHM-15-0-s002.docx]

Supporting Information

Electrospun Core–Shell Bioadhesive Nanofiber Sheets Enable Durable Pulmonary Air Leak Sealing under Wet and Dynamic Conditions

Tomohito Okubo, Kouji Hara, Naoyuki Hatayama, Kota Takahashi, Eriko Yamazoe, Takaaki Ito, Takao Takeuchi, Yoko Koide, Mayumi Yamada, Munekazu Naito, Kohei Tahara^＊^

**Figure S1.** Scanning electron microscopy images used for quantitative morphological analysis.

SEM images acquired at ×2,000 magnification are shown for A) base, B) hybrid-PVA, C) core–shell, D) emulsion, E) layer-by-layer PCL outer side, and layer-by-layer PVA/CMC-Na tissue-contacting side. One image was analyzed for each sheet type or surface. Images acquired at ×2,000 magnification were used because their wider field of view contained more fibers than the ×5,000 images shown in Figure 3. Scale bars: 10 µm.

**Figure S2.** Segmentation of fibers and pores in the SEM images used for quantitative morphological analysis.

The SEM images shown in Figure S2 were segmented using MIPAR software for A) base, B) hybrid-PVA, C) core–shell, D) emulsion, E) layer-by-layer PCL outer side, and layer-by-layer PVA/CMC-Na tissue-contacting side. Fibers are shown in cyan, and pore regions are shown in green. The segmented images were used to calculate fiber diameter and the area fractions of fibers and pores.

**Figure S3.** Confocal laser scanning microscopy images of the core–shell nanofiber sheet used for colocalization analysis (Images 1–5). Columns from left to right show coumarin-6 fluorescence associated with the PCL phase (green), sulforhodamine B fluorescence associated with the PVA/CMC-Na phase (red), bright-field images, and merged images. All images were analyzed using the Coloc 2 plugin in Fiji/ImageJ. Thresholded Manders’ coefficients (tM1 and tM2) were calculated after Costes automatic thresholding to semi-quantitatively evaluate the spatial colocalization of the two polymer-associated fluorescence signals. Scale bars: 50 μm.

**Figure S3 (continued).** Confocal laser scanning microscopy images of the core–shell nanofiber sheet used for colocalization analysis (Images 6–10).

**Figure S4.** Quantitative air leak data over the 1-h periods before and after treatment for all subjects (*N* = 5). Each data point represents average air leak volume over a 10-min interval (e.g., the data point at 10 min before treatment indicates the average leak volume from 0 min to 10 min after lung injury induction). The average air leak volume of the five subjects during the 1-h pretreatment period was 51.4 mL/min, with values of 67.0 mL/min at 0–10 min, 52.8 mL/min at 10–20 min, 49.2 mL/min at 20–30 min, 48 mL/min at 30–40 min, 45.2 mL/min at 40–50 min, and 46.2 mL/min at 50–60 min. During 1-h posttreatment, average air leak volume decreased to 3.6 mL/min, with values of 5.6 mL/min at 0–10 min, 3.0 mL/min at 10–20 min, 3.0 mL/min at 20–30 min, 1.8 mL/min at 30–40 min, 6.4 mL/min at 40–50 min, and 1.8 mL/min at 50–60 min. In one subject, posttreatment air leak volume at the 40–50 min interval increased due to bucking—an involuntary respiratory effort against the endotracheal tube—causing air to escape from the thoracic cavity and be recorded as an apparent leak.

**Table S1.** Evaluation criteria for nanofiber sheet performance in pulmonary fistula closure. Performance was assessed using three parameters: vertical rupture resistance, lateral leakage resistance, and seal retention under wet conditions. Each parameter was scored on a five-point scale (1–5), with higher scores indicating superior performance. The balance score was calculated from the average of the three individual scores. To avoid overestimating sheets with marked deficiency in any single performance domain, the maximum balance score was limited to 3 whenever any of the three parameters received a score of ≤2. The overall score was calculated by multiplying the sum of the three individual scores by the balance score.

| Evaluation item | Score | Criteria |
| --- | --- | --- |
| Vertical rupture resistance  (VRR) | 1 | Perforation at peak airway pressure < 20 cmH_2_O |
|  | 2 | 20 cmH_2_O ≤ pressure < 25 cmH_2_O |
|  | 3 | 25 cmH_2_O ≤ pressure < 30 cmH_2_O |
|  | 4 | Perforation at a pressure of 30 cmH₂O |
|  | 5 | Withstands pressure of 30 cmH₂O |
| Lateral leakage resistance  (LLR) | 1 | Leakage at peak airway pressure < 20 cmH_2_O |
|  | 2 | 20 cmH_2_O ≤ pressure < 25 cmH_2_O |
|  | 3 | 25 cmH_2_O ≤ pressure < 30 cmH_2_O |
|  | 4 | Leakage at a pressure of 30 cmH₂O |
|  | 5 | Withstands a pressure of 30 cmH₂O |
| Seal retention under wet conditions  (SRW) | 1 | Fails in pleural fluid only |
|  | 2 | Immediate failure with saline drop |
|  | 3 | Failure within 30 min after saline drop |
|  | 4 | Failure within 60 min after saline drop |
|  | 5 | Withstands > 60 min after saline drop |
| Average score | Balance score | Interpretation |
| ≤2.0 | 1 | Severely imbalanced |
| 2.0 < Average ≤ 2.8 | 2 | Slightly imbalanced |
| 2.8 < Average ≤ 3.6 | 3 | Balanced within the acceptable range |
| 3.6 < Average ≤ 4.4 | 4 | Well balanced within the acceptable range |
| > 4.4 | 5 | Ideally balanced |
| Overall score = (VRR + LLR + SRW) × balance score | | |

**Table S2.** Semi-quantitative morphological characteristics of electrospun nanofiber sheets determined by MIPAR image analysis. Fiber diameter and the area fractions occupied by fibers and pores were calculated from SEM images acquired at ×2,000 magnification (Figure S1 and S2). One SEM image was analyzed for each sheet type or surface, and all fibers detected within the field of view were included in the automated analysis. Fiber diameter values are presented as the mean ± standard deviation of the fibers detected in the analyzed image. Fiber and pore area fractions are expressed as percentages of the total analyzed image area. For the layer-by-layer sheet, the PCL outer side and PVA/CMC-Na tissue-contacting side were analyzed separately.

| Sheet type |  | Fiber diameter (nm) | Area Fraction (%) | |
| --- | --- | --- | --- | --- |
|  |  |  | Fibers | Pores |
| Base |  | 445 ± 179 | 49.6 | 50.4 |
| Hybrid-PVA |  | 567 ± 209 | 51.4 | 48.6 |
| Core–Shell |  | 429 ± 190 | 50.7 | 49.3 |
| Emulsion |  | 446 ± 226 | 49.8 | 50.2 |
| Layer-by-Layer | PCL (Outer side) | 666 ± 369 | 53.0 | 47.0 |
|  | PVA/CMC-Na (Tissue side) | 459 ± 184 | 54.5 | 45.5 |

**Table S3.** Semi-quantitative colocalization analysis of the core–shell nanofiber sheet by confocal laser scanning microscopy. Thresholded and unthresholded Manders' colocalization coefficients were calculated for ten CLSM fields (Images 1–10) using the Coloc 2 plugin in Fiji/ImageJ. Coumarin-6 fluorescence associated with the PCL phase was assigned to channel 1, and sulforhodamine B fluorescence associated with the PVA/CMC-Na phase was assigned to channel 2. Thresholded coefficients (tM1 and tM2) were calculated after Costes automatic thresholding and were used for the semi-quantitative evaluation of the spatial colocalization of the two polymer-associated fluorescence signals. Data are presented as individual values for each image, together with the mean and standard deviation (SD).

It should be noted that Manders' coefficients quantify fluorescence colocalization rather than the numerical proportion of individual fibers exhibiting a true concentric core–shell structure.

| Image No. | Manders' M1 | Manders' M2 | Manders' tM1 | Manders' tM2 |
| --- | --- | --- | --- | --- |
| 1 | 0.94 | 0.90 | 0.40 | 0.29 |
| 2 | 0.82 | 0.87 | 0.59 | 0.39 |
| 3 | 0.56 | 0.90 | 0.49 | 0.69 |
| 4 | 0.78 | 0.85 | 0.60 | 0.44 |
| 5 | 0.74 | 0.39 | 0.35 | 0.23 |
| 6 | 0.94 | 0.70 | 0.63 | 0.33 |
| 7 | 0.87 | 0.89 | 0.76 | 0.67 |
| 8 | 0.85 | 0.83 | 0.72 | 0.60 |
| 9 | 0.97 | 0.94 | 0.49 | 0.25 |
| 10 | 0.57 | 0.84 | 0.45 | 0.52 |
| Mean ± S.D. | 0.80 ± 0.14 | 0.81 ±0.16 | 0.55 ± 0.14 | 0.44 ± 0.17 |

**Table S4.** Mechanical properties of electrospun nanofiber sheets obtained from tensile testing under dry conditions (corresponding to Figure 5A). Values are presented as mean ± standard deviation (n = 4). Toughness was calculated from the area under the stress–strain curve.

| Sample | Young's modulus (MPa) | Tensile Strength (MPa) | Toughness (MJ/m³) |
| --- | --- | --- | --- |
| Base | 255.3 ± 19.1 | 5.52 ± 0.38 | 1.84 ± 0.24 |
| Hybrid-PVA | 275.5 ± 64.5 | 9.38 ± 0.31 | 13.26 ± 2.13 |
| Core-Shell | 281.5 ± 45.6 | 8.08 ± 0.60 | 3.51 ± 1.05 |
| Emulsion | 147.3 ± 7.9 | 4.04 ± 0.06 | 0.64 ± 0.13 |
| Layer-by-Layer | 255.4 ± 13.3 | 5.84 ± 0.35 | 3.56 ± 2.45 |

**Table S5.** Mechanical properties of electrospun nanofiber sheets obtained from tensile testing under hydrated conditions (corresponding to Figure 5B). Values are presented as mean ± standard deviation (n = 4). Toughness was calculated from the area under the stress–strain curve. N.D. indicates that the specimens lost their structural integrity immediately after wetting and therefore could not be subjected to tensile testing.

| Sample | Young's modulus (MPa) | Tensile Strength (MPa) | Toughness (MJ/m³) |
| --- | --- | --- | --- |
| Base | N.D. | N.D. | N.D. |
| Hybrid-PVA | 1.020 ± 0.291 | 0.403 ± 0.047 | 0.030 ± 0.013 |
| Core-Shell | 0.310 ± 0.112 | 0.041 ± 0.004 | 0.0096 ± 0.0035 |
| Emulsion | N.D. | N.D. | N.D. |
| Layer-by-Layer | 0.240 ± 0.153 | 0.522 ± 0.079 | 2.08 ± 0.35 |
